# Developing and evaluating image-based metrics for open surgical suturing skill assessment

**DOI:** 10.1007/s44186-026-00596-2

**Published:** 2026-10-13

**Authors:** Jianxin Gao, Simar P. Singh, Amir Mehdi Shayan, John F. Eidt, Joe Bible, Joseph Vincent Valentino Blas, Sagar S. Gandhi, Ravikiran Singapogu, Richard E. Groff

**Affiliations:** 1 Department of Electrical and Computer Engineering, Clemson University, Clemson, SC USA; 2 Department of Bioengineering, Clemson University, Clemson, SC USA; 3 Division of Vascular Surgery, Baylor Scott & White Heart and Vascular Hospital, Dallas, TX USA; 4 School of Mathematical and Statistical Sciences, Clemson University, Clemson, SC USA; 5 School of Medicine, University of South Carolina, Greenville, SC USA

**Keywords:** Computer vision, Surgical skill assessment, Objective metrics, Medical simulator, Open surgery, Suturing

## Abstract

**Purpose:**

Open surgery training is vital in vascular surgery education. Traditionally, trainees’ open surgical suturing skills are evaluated by their educators, which is a subjective and time-consuming process. This paper evaluates image-based metrics for surgical suturing skill assessment by comparing performance among three groups: attending surgeons, resident surgeons, and novices.

**Methods:**

A simulator, named SutureCoach, is developed to simulate a radial suturing task, performed either at “depth,” representing a cavity, or at “surface.” Among other sensors, the simulator includes cameras and computer vision software to track the motion of the needle and thread under the membrane being sutured. A set of image-based metrics are calculated for each suture based on video data. The set of metrics includes four previously proposed metrics as well as four new metrics inspired by a refined suturing phase segmentation scheme. To evaluate the performance of the image-based metrics, we conducted a study with 97 participants, divided almost evenly between attending surgeons, resident surgeons, and novices. Data was collected at several events over a 5-month period.

**Results:**

The statistical analysis shows that all metrics have significantly different means when comparing performance between novices and surgeons at both conditions, surface and depth. Furthermore, seven out of eight metrics have significant mean differences when comparing resident surgeons’ and attending surgeons’ performance at the depth condition.

**Conclusion:**

Image-based metrics are capable of suturing skill assessment, especially for distinguishing novices from surgeons. Depth constraints allow higher skill levels to be better differentiated.

**Supplementary Information:**

The online version contains supplementary material available at https://doi.org/10.1007/s44186-026-00596-2.

## Introduction

Due to the rapid development of endovascular surgery, conditions originally treated by open surgery are shifting to endovascular interventions. As a result, trainees encounter open surgery less often. Nevertheless, there are still complex circumstances that require open surgical techniques in vascular surgery [1]. To address the training challenges, educators have developed a simulation-based examination called Fundamentals of Vascular Surgery (FVS) [1, 2].

Suturing is considered a fundamental skill in vascular training [1], as complications related to poor suturing can lead to prolonged hospitalization [3]. Also, research shows that suturing skills can be used to identify trainees’ capabilities [4]. The FVS includes a clock-face model for evaluating radial suturing skill [1], but scoring relies on direct observation by trained evaluators, which is subjective and time-consuming [1, 5]. By instrumenting the simulator, skill assessment metrics can be calculated from quantitative sensor data.

The research community is pursuing sensor-embedded simulators for open surgical training, which provide objective performance assessment [6] and obviate the need for manual scoring [7, 8]. Research suggests that skills learned on a bench model can be transferred to surgical settings [9, 10]. Several simulators have been developed to evaluate open surgical skills [11–15]. Simulators with electromagnetic trackers can track hand motions and record task duration [11–13]. Some simulators are equipped with a force sensor under the suturing platform, which measure the force applied to the platform by the participant during performance [14, 15]. These simulators are proven to be promising in distinguishing participants’ skill levels in open surgery.

In order to objectively quantify performance, metrics are calculated based on data recorded from one or more sensors. For example, there are metrics based on hand or tool motion [11–14, 16–21], based on measured forces [14, 22–25], and based on video from cameras [26–30]. Motivated by input from surgeons, metrics are formulated to capture features of the sensor data related to surgical skill. As an alternative approach, recent studies have proposed using deep-learning models to analyze suturing videos without specifying the metrics for skill assessment [31–35]. Although the deep learning models demonstrate high accuracy for classifying participants’ skill levels, they have not been widely used in surgical training due to the lack of physical interpretations of the model outputs. Also, our literature review shows that few simulators use video analysis to compute metrics for evaluating suturing skills in open surgery tasks.

**Fig. 1:**
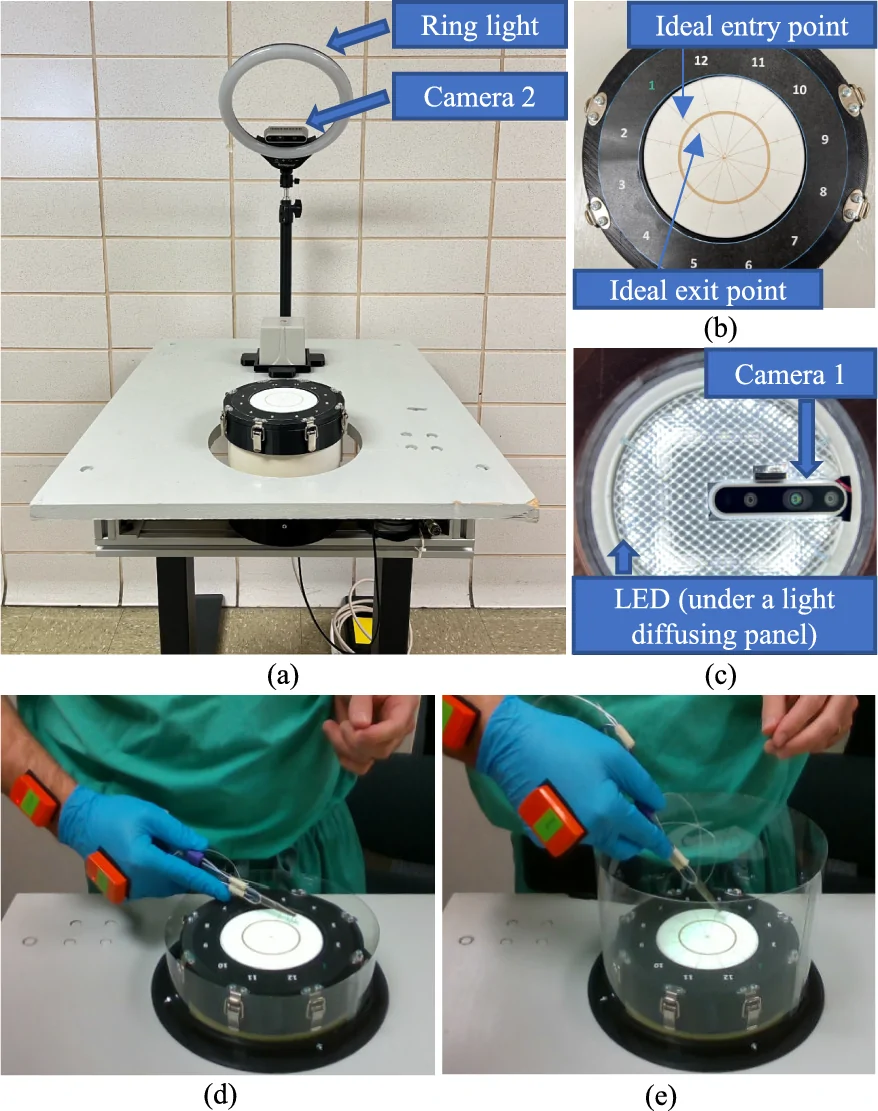
The suturing simulator: **a** Front view; **b** Top view of the membrane housing; **c** The internal structure of the membrane housing; **d** Surface condition; **e** Depth condition

SutureCoach is an instrumented simulator based on the radial suturing task from FVS. The design of SutureCoach is inspired by an earlier prototype [36], used in a pilot study on image-based metrics [37–39]. The SutureCoach simulator includes two cameras, two electromagnetic sensors attached to the tool, two inertial measurement units attached to the wrist and the back of hand, and a force sensor at the base of the membrane holder. Compared to the prototype, SutureCoach improves portability by simplifying the table structure (see Fig. 1a) and the depth constraint (see Fig. 1d and e). The membrane housing and light sources were redesigned and the computer vision algorithms were reworked to improve reliability. The simulator software was redeveloped to improve modularity, making it easier to add new sensors or collect from a subset of sensors. The improvements in mobility and robustness enabled the large study presented here.

SutureCoach was used to collect data from almost one hundred subjects, including over 60 surgeons. This paper focuses on the effectiveness of image-based metrics for surgical suturing skill comparison among attending surgeons, resident surgeons, and novices. Metrics based on the other sensors were reported separately [19–21, 25, 40]. The image-based metrics are based on video from the camera located under the suturing membrane (see Camera 1 in Fig. 1c) and do not require sensors to be mounted on surgical tools or hands. It is hypothesized that the proposed image-based metrics have different statistical results among attending surgeons, resident surgeons, and novices.

This paper is organized as follows. The first half of “Materials and methods” covers model development, divided into subsections on improvements to the SutureCoach “Simulator”, the “Suture phase segmentation” scheme, “Skill assessment metrics from previous works” and “Skill assessment metrics based on suture phases”. The second half of “Materials and methods” covers model evaluation, including “Data collection” and “Comparing metric performance". The “Results” section provides results and statistical analysis. In “Discussion”, the classification performance using image-based metrics is discussed. Finally, conclusions and future work are in “Conclusion”.

## Materials and methods

### Simulator

SutureCoach [19] is designed for measuring suturing skills on the clock face model of FVS. Design changes were made to improve the portability and reliability of SutureCoach in comparison to the previous version of the simulator [36], in order to allow consistent data collection in a variety of environments. The changes focused on the table frame, the vertical constraints, and the object tracking system. SutureCoach is based on a height-adjustable table (see Fig. 1a) chosen to be heavier than the previous version [36], providing stability without the use of an additional aluminum barrier. SutureCoach simulates two suturing conditions, surface and depth, using cylindrical vertical constraints (see Fig. 1d and e). The constraints are clear cylindrical PETG sheets inserted into a 3D printed base. Suturing conditions can be quickly changed by swapping out the PETG cylinders, significantly simplifying the motorized height-adjustment mechanism of the previous system [36]. SutureCoach has a 3D-printed membrane holder (See Fig. 1b and c) and two Intel RealSense D435 cameras. The camera in the membrane holder (i.e., Camera 1 in Fig. 1c) records RGB videos with 640*× *480 resolution at 60 frames per second (fps). An LED strip and a light diffusing panel are installed to guarantee a stable light source in the membrane holder, which improves the reliability of the computer vision. The camera mounted above the membrane holder (i.e., Camera 2 in Fig. 1a) records both RGB and depth videos with 848*× *480 resolution at 30 fps. It is equipped with a ring light to provide consistent lighting.

SutureCoach processes sensor data by a desktop computer with an Intel Xeon CPU and an NVIDIA Quadro RTX 4000 GPU. Its data processing strategy includes (i) the data-collection stage, and (ii) the data-analysis stage. In stage (i), two C++ programs, written on MS Visual Studio 2017 configured with OpenCV CUDA module, are used for data collection and object tracking. Specifically, the data-collection program is used to synchronize and record data from multiple sensors, namely two cameras, two EM position sensors, two inertial measurement units, and a force sensor. Data synchronization allows us to concurrently study multiple signals and identify erroneous movement [41]. While collecting data, the program simulateously sends the video stream from Camera 1 to the computer vision program, which supports tracking the needle and thread in real-time [39]. Compared with the code in [39], the two C++ programs use an improved data management scheme and have higher fault tolerance. For example, the data-collection program automatically assigns each participant a hash ID, which is then used to organize the data. Furthermore, the sensor data would be saved without any loss even if the computer vision program fails unexpectedly. In stage (ii), a custom Python program is used to calculate the skill-assessment metrics by analyzing the output from the computer vision program.

**Fig. 2:**
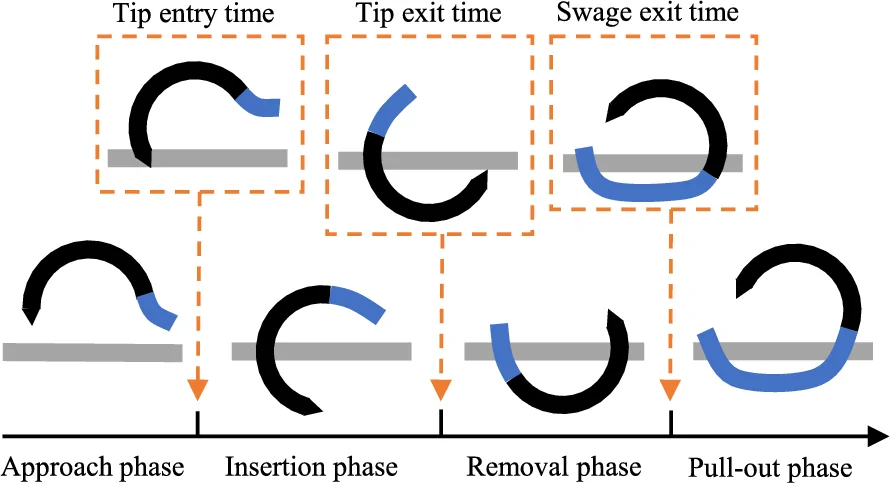
Suture phase segmentation. The needle is denoted by the black semi-circles, while the thread is denoted by the blue curves

### Suture phase segmentation

The general suture rules in [42–44] divide the suturing process into several essential steps, motivating us to improve the suture phase segmentation scheme introduced in [45]. In addition to the tip entry and swage exit times calculated in previous works [36, 37, 45], the software now also calculates tip exit time, so that the four suture phases (see Fig. 2) can be automatically segmented by the computer vision program. The refined segmentation allows sensor data to be extracted for specific phases of the suture, which helps interpret data and evaluate participants’ performance.

As shown in Fig. 2, the suture cycle is divided based on tip entry time, tip exit time, and swage exit time. For each suture, the tip entry time corresponds to the first video frame in which the needle is detected, while the swage exit time corresponds to the last video frame in which the needle is detected.

The tip exit time is defined as when the needle tip starts piercing the membrane in an upward direction. Its calculation requires the needle exit point and the needle tip trajectory detected by the computer vision program. By calculating the distance between the needle exit point and the points on the needle tip trajectory, the tip exit time is chosen as the timestamp corresponding to the last needle tip point that is 1*mm* away from the needle exit point.

After the segmentation, the suture cycle is split into 4 phases.

- Approach phase is the period before the needle punctures the membrane.
- Insertion phase is the period when a participant inserts the needle body into the membrane housing and then moves the needle tip toward the exit point.
- Removal phase is the period when a participant re-grasps the needle tip and then removes the needle body from the membrane housing.
- Pull-out phase is the period for pulling out the thread.

### Skill assessment metrics from previous works

Four of the image-based metrics [38, 39] developed for the suturing simulator prototype [36, 39] are also used to analyze the SutureCoach dataset.

*Needle Tip Path Length* (TipPathLen) is the length of the needle tip trajectory during the insertion phase. The metric is to measure participants’ motion economy. Before calculating TipPathLen, the noise-removal techniques in [39] were adopted to filter the detected tip trajectory. The metric is then calculated by

1 $$\begin{aligned} \texttt {TipPathLen}=k_{cam} \sum _{i=1}^{n-1} || p_{i+1} - p_{i} ||_2 \end{aligned}$$

where $k_{cam}$ denotes the pixel-to-mm conversion ratio (i.e., 0.21 for SutureCoach), $p_i$ denotes the $i^{th}$ filtered tip point in Cartesian coordinate system, and *n* denotes the number of filtered tip points.

*Needle swept area* (SweptArea) is the accumulated needle locations during insertion and removal phases. To calculate the metric, the computer vision program detects the needle per video frame from Camera 1. After each suture, SweptArea is area of the union of the detected needle locations, expressed in units of $mm^2$. SweptArea is calculated by

2 $$\begin{aligned} \texttt {SweptArea}=k^2_{cam}*n \end{aligned}$$

where *n* denotes the number of pixels in the binary image of accumulated needle locations.

*Needle sway length* (SwayLen) measures the needle rotation about the axis from the needle entry point to the needle tip. To calculate SwayLen for frame *i*, the entry point is virtually connected to the tip. An orthogonal line is then drawn at the midpoint of the connection. The distance, from the midpoint to the intersection of the orthogonal line and the needle body, is defined as $\texttt {SwayLen}_{i}$. Suppose insertion and removal phases of a suture contain *n* frames in total, SwayLen is calculated by

3 $$\begin{aligned} \begin{aligned} \texttt {SwayLen}=&\max _{i} \{ \texttt {SwayLen}_{1,...,n} \} \\&-\min _{i} \{ \texttt {SwayLen}_{1,...,n} \} \end{aligned} \end{aligned}$$

*Stitch length* (StitchLen) is the distance between needle entry point $p_{en}$ and needle exit point $p_{ex}$. This metric has been used to analyze participants’ performance for closing a straight incision on a foam skin pad [26]. StitchLen is obtained by

4 $$\begin{aligned} \texttt {StitchLen}= k_{cam} || p_{en} - p_{ex} ||_2 \end{aligned}$$

### Skill assessment metrics based on suture phases

Using the refined suture phase segmentation scheme, the suturing skill can be further evaluated by four metrics, namely needle angle range (Range(NAngle)), average needle angle (AVG(NAngle)), range of orthogonal tip projection (Range(TipOrth)), and duration for needle removal (RemovalTime). Since Range(NAngle), AVG(NAngle), and Range(TipOrth) are affected by tip detection accuracy, the filtered tip points for TipPathLen calculation are used. Notice that Range(NAngle), AVG(NAngle), and Range(TipOrth) evaluate the participant’s performance during the insertion phase, while RemovalTime evaluates the performance during the removal phase.

**Fig. 3:**
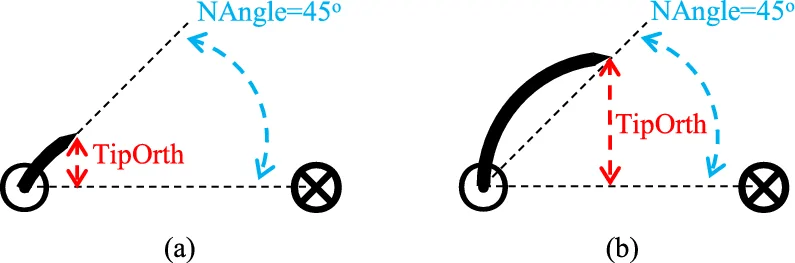
NAngle versus TipOrth. **a** At the beginning of the insertion phase; **b** At the middle of the insertion phase. Notice that **a** and **b** has identical NAngle but different TipOrth. The needle, the entry point, and the exit point are denoted by the black curve, *⨀ *, and *⨂ *, respectively

Range(NAngle) measures the range of needle lateral movement in the form of rotation, where the needle angle (NAngle) is defined as the angle between the tip-to-entry connection and the entry-to-exit connection in a single video frame (see Fig. 3). A participant with a small Range(NAngle) is expected to have high suturing skill, as the best sutures involve an insertion perpendicular to the tissue surface and a needle path following the curvature of the needle [42, 46].

Equation 5 is for NAngle calculation. Specifically, $v_{tip}$ denotes the vector pointing from the entry point to the needle tip, while $\hat{e}_{tan}$, and $\hat{e}_{orth}$ are the tangential and the orthogonal unit vector for the entry-to-exit connection, respectively.

5 $$\begin{aligned} \texttt {NAngle}={{\,\textrm{atan2}\,}}(v_{tip} \cdot \hat{e}_{orth}, v_{tip} \cdot \hat{e}_{tan}) \end{aligned}$$

Suppose the insertion phase of a single suture contains *n* frames, Range(NAngle) is then determined by

6 $$\begin{aligned} \begin{aligned} \texttt {Range(NAngle)}=&\max _i \{\texttt {NAngle}_{1,...,n}\} \\&- \min _i \{\texttt {NAngle}_{1,...,n}\} \end{aligned} \end{aligned}$$

AVG(NAngle) is the mean of absolute NAngle during the insertion phase. The metric is expected to be inversely proportional to the experience levels. AVG(NAngle) is calculated by

7 $$\begin{aligned} \texttt {AVG(NAngle)}=\frac{\sum _{i=1}^{n} |\texttt {NAngle}_i|}{n} \end{aligned}$$

where *n* denotes the number of video frames during the insertion phase.

Range(TipOrth) measures the range of needle lateral movement in the form of projection, where the orthogonal tip projection (TipOrth) is the projection of the tip-to-entry connection to $\hat{e}_{orth}$ in a single video frame. A small Range(TipOrth) implies high suturing skill.

Compared with NAngle, TipOrth requires higher detection accuracy of the needle tip, but it is more sensitive to needle deviation when the needle tip approaches the exit point. An illustration is shown in Fig. 3. It is noticeable that the needle lateral movement is significantly larger in Fig. 3b than in Fig. 3a. The difference between NAngle in Fig. 3a and b, however, is much smaller than the difference between TipOrth in the two subfigures.

Equation (8) is for TipOrth calculation, where $v_{tip}$ denotes the vector pointing from the entry point to the needle tip, and $\hat{e}_{orth}$ represents the orthogonal unit vector for the needle entry-to-exit connection.

8 $$\begin{aligned} \texttt {TipOrth}=v_{tip} \cdot \hat{e}_{orth} \end{aligned}$$

Suppose the insertion phase of a single suture contains *n* frames, Range(TipOrth) is then determined by

9 $$\begin{aligned} \begin{aligned} \texttt {Range(TipOrth)}=&\max _i \{\texttt {TipOrth}_{1,...,n}\} \\&- \min _i \{\texttt {TipOrth}_{1,...,n}\} \end{aligned} \end{aligned}$$

RemovalTime is how long a participant spends in the removal phase, which is calculated by

10 $$\begin{aligned} \texttt {RemovalTime}=T_{swage\; exit} - T_{tip\; exit} \end{aligned}$$

where $T_{swage\; exit}$ and $T_{tip\; exit}$ denote the swage exit time and the tip exit time, respectively.

### Data collection

SutureCoach data were collected over a 5 month period across multiple settings, including a university campus, a hospital, and several vascular conferences. Because this study aimed to evaluate the ability of SutureCoach metrics to distinguish between levels of suturing skill, we recruited surgical trainees and attending surgeons from these settings. This recruitment strategy enabled us to obtain a larger, multi-institutional, and geographically diverse sample, although it is not without limitations (discussed in the Conclusions section). Detailed demographic information is provided in Table 1 of [19]. Before data collection, all participants received standardized instructions on simulator use.

During data collection, each participant was required to perform four trials on SutureCoach: The first trial was at surface condition (see Fig. 1d), followed by a trial at depth condition (see Fig. 1e). Then the participant repeated the two trials in the same order. In each trial, the participant performed outside-in sutures, starting at Area 1 (see Fig. 1b), and proceeding in a counter-clockwise direction. The suturing pattern (see Fig. 1b) is based on the FVS clock face model [1, 47]. All trials used the 3-0 Prolene sutures (26 mm 1/2c SH needle).

The dataset includes 97 participants separated into three groups:

- The expert group consists of 35 participants who are fellows or attending surgeons.
- The intermediate group consists of 32 resident surgeons whose postgraduate year (PGY) is from one to five.
- The novice group consists of 30 participants who are medical students or other students.

**Fig. 4:**
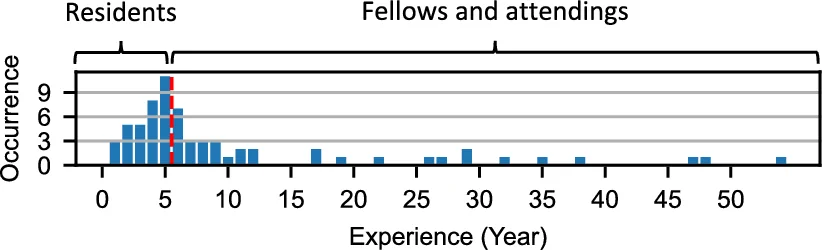
Distribution for the resident surgeons, fellows, and attending surgeons’ year of experience. The intermediate group is on the left of the red dashed line, while the expert group is on the right of the red dashed line

Figure 4 shows the distribution of years of experience in expert and intermediate groups. In Fig. 4, a first-year fellow is considered to have six years of experience, while a first-year attending surgeon is considered to have eight years of experience. The red dashed line in Fig. 4 denotes the boundary between intermediate and expert groups. Notice that the novice group is considered to have zero years of experience.

For our dataset, expert, intermediate, and novice groups have 1695, 1509, and 1500 detected sutures, respectively. Some of the suture data (less than *0.5%*) are removed from the dataset, as participants finished the sutures without puncturing the membrane material. This situation caused the computer vision program to have inaccurate needle and thread detection.

### Comparing metric performance

The first statistical question examined is whether the metric values for the three groups have different means. Since the dataset contains a large number of individual sutures and the metric values have heterogeneous variances for each group, this paper uses one-way analysis of variance (one-way ANOVA) and Welch’s t-test to answer this question. If *p<0.05*, the group means are considered to be different.

One-way ANOVA and Welch’s t-test examine whether the metric performance of the three groups is different, but the ultimate goal of skill assessment is to classify the performance of individual participants. Receiver Operating Characteristics (ROC) curves are specifically designed to visualize the performance of two-group threshold-based classifiers and thus are useful for comparing the effectiveness of individual metrics for use in skill assessment. Specifically, an ROC curve shows how the true positive rate (TPR) and the false positive rate (FPR) vary as the classifier threshold value is varied.

The metric classification performance is evaluated by the ROC curves for the suture classifier and for the trial classifier. For the suture classifier, a participant’s performance is determined based on a single suture. The trial classifier, however, determines a participant’s performance by the median metric value of a single trial. Compared with the suture classifier, the trial classifier is expected to have higher performance, since the decision is based on more measurements. Specifically, the median summarizes the participant’s performance on the 12 sutures of the trial. Further, the median is insensitive to extreme values that might arise due to sensor noise.

A popular way to evaluate the performance of a classifier is to measure the area under its ROC curves, known as area under curve (AUC). AUC is considered to be a measure of aggregate classification performance, as an ROC curve includes TPR and FPR determined by all possible decision thresholds [48]. For example, a random classifier has AUC equal to 0.5, since its ROC curve is the diagonal from (0, 0) to (1, 1). In contrast, AUC for an ideal classifier is equal to 1, as the corresponding ROC curve approaches the top-left corner of the ROC graph [49].

## Results

For the eight metrics, one-way ANOVA shows that the means of the three groups are significantly different at both surface and depth conditions.

**Fig. 5:**
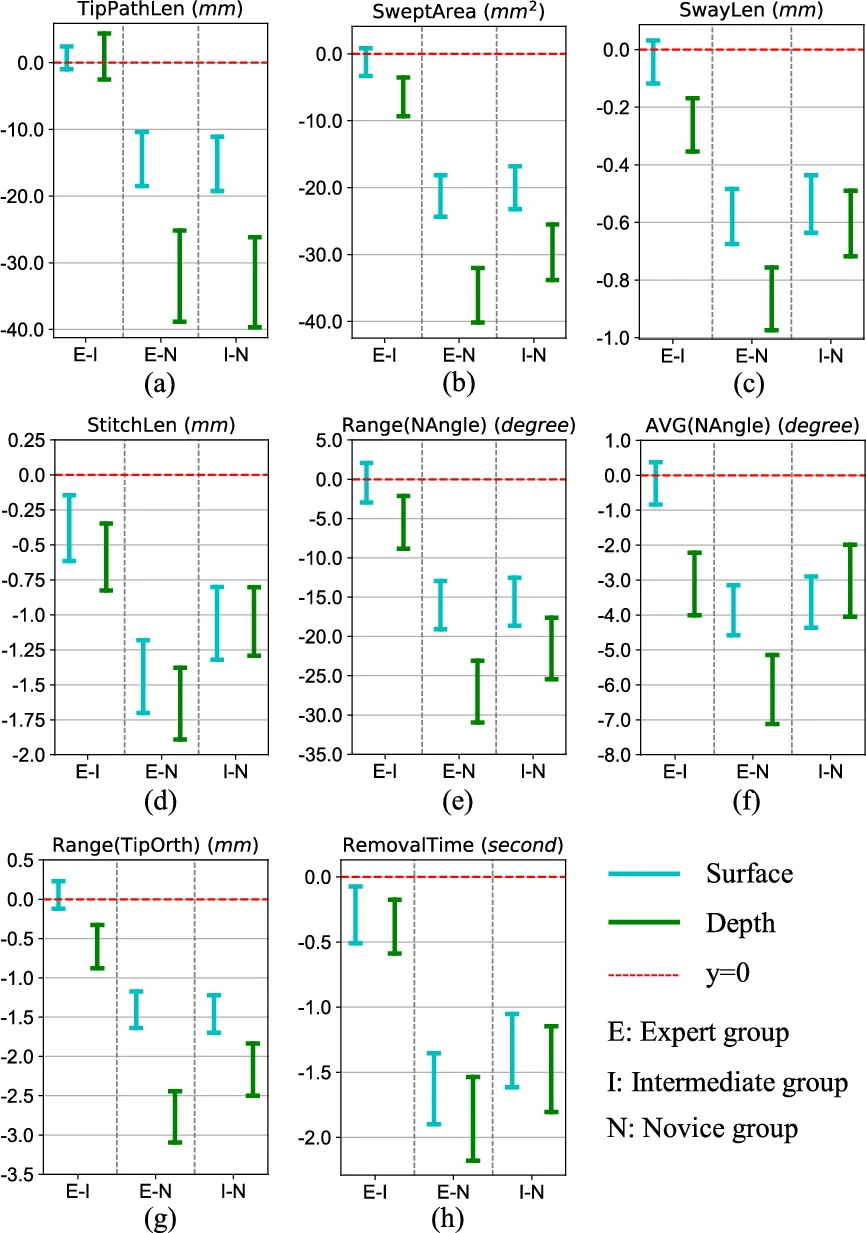
Confidence intervals for the eight metrics showing pairwise comparisons of experts versus intermediates (E-I), experts versus novices (E-N), and intermediates versus novices (I-N). For each pairwise comparison, surface and depth conditions are shown separately

The confidence intervals calculated by Welch’s *t*-test are shown in Fig. 5, where each subfigure shows the comparisons of experts versus intermediates (E-I), experts versus novices (E-N), and intermediates versus novices (I-N) for a specific metric. The comparisons are made separately for surface and depth conditions. The confidence interval denotes the range of likely differences between the two group means. Therefore, if a confidence interval does not cross zero, indicated by the red dashed line, then the null hypothesis is rejected, i.e. there is a statistically significant difference between the mean of the two groups. Further statistics for the metrics are provided in Supplementary Table S1 in Online Resource 1.

**Fig. 6:**
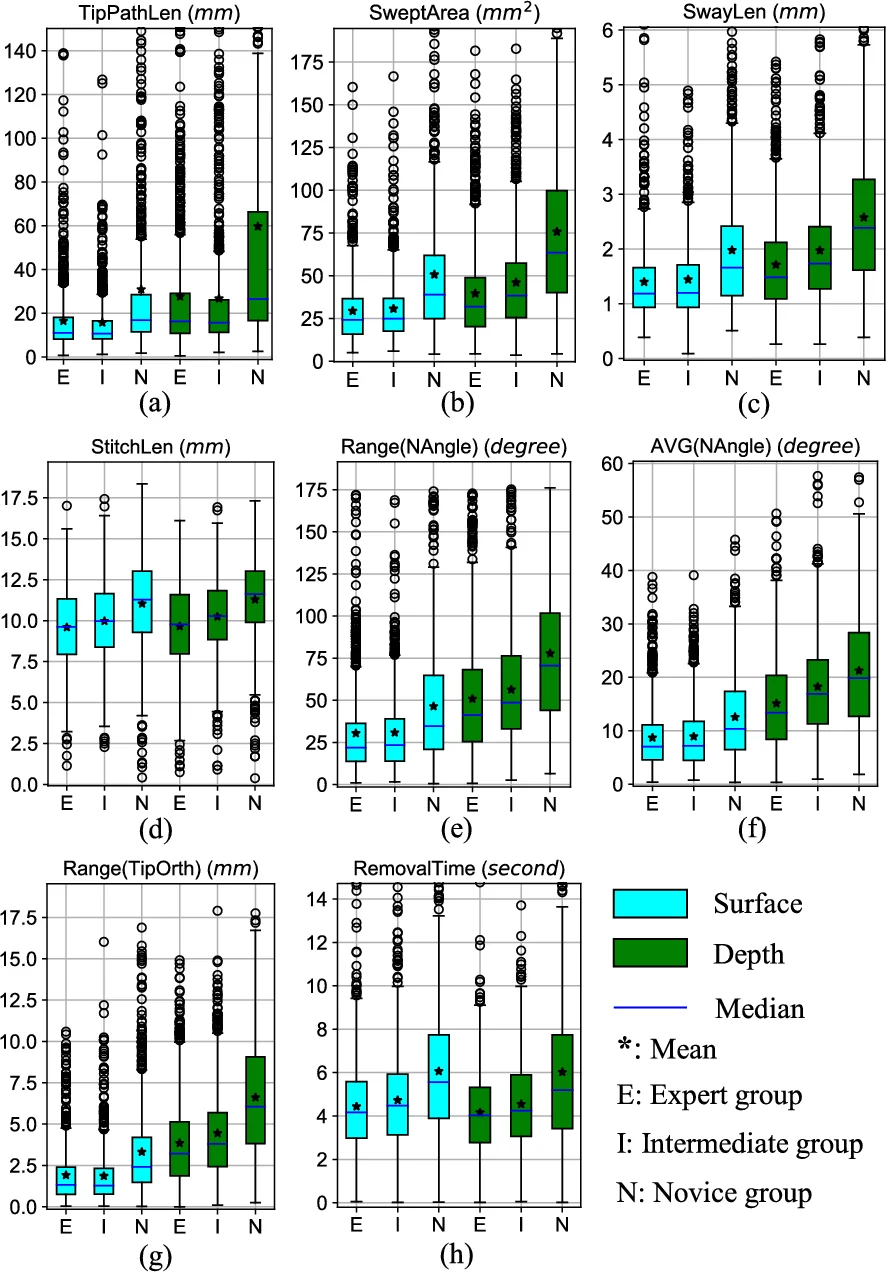
Boxplots of the metric values for the three groups. Each subfigure presents the outputs of a single metric at surface and depth conditions. The extreme outliers are not shown in the boxplots

Boxplots for the eight metrics are shown in Fig. 6, where metric values for the three groups are presented at the surface and depth conditions separately. Figure 6 also includes the mean metric values for each group. Extreme outliers are not shown in Fig. 6 to avoid cluttering the figures.

**Fig. 7:**
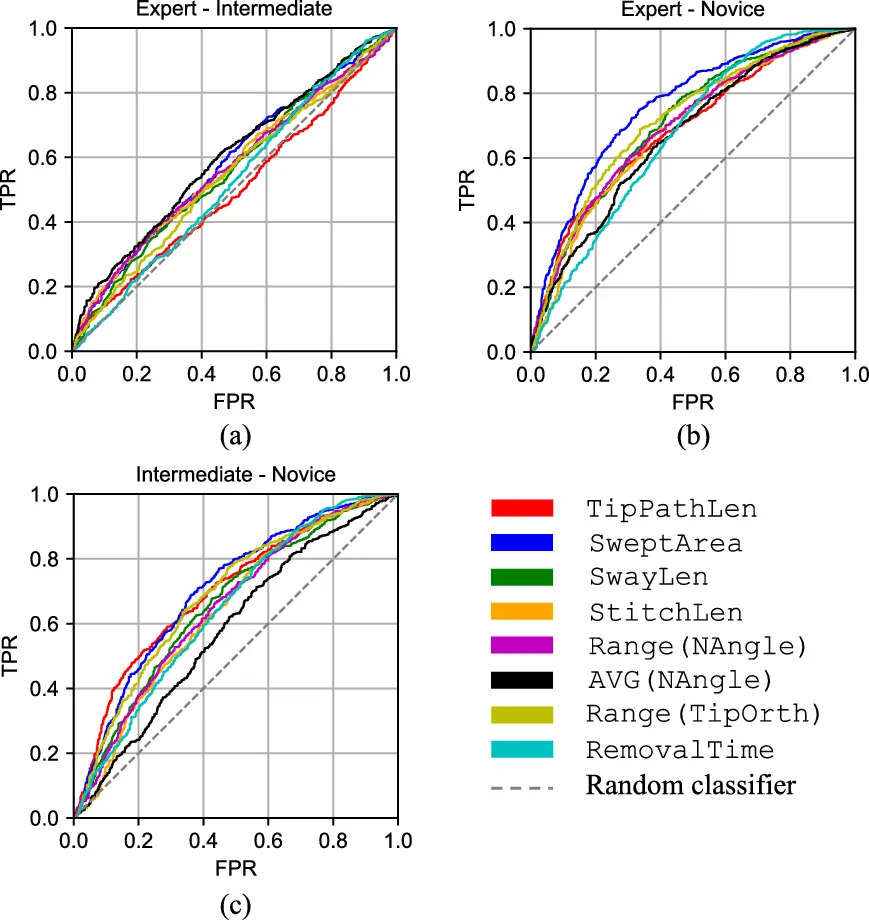
ROC curves for the suture classifier. Each subfigure shows the classification between two groups at the depth condition

**Fig. 8:**
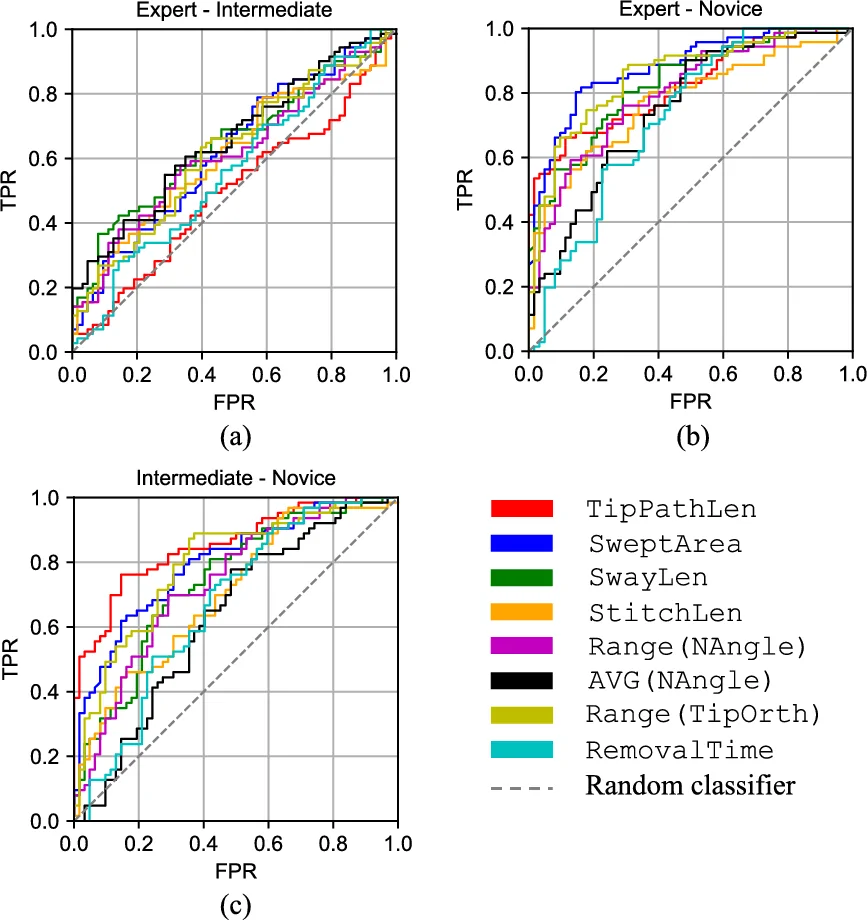
ROC curves for the trial classifier. Each subfigure shows the classification between two groups at the depth condition

Figure 7 and Fig. 8 present ROC curves for the eight metrics at the depth condition. Specifically, Fig. 7 shows the ROC curves for the suture classifier, while Fig. 8 shows the ROC curves for the trial classifier. In the two figures, the group with more experience is considered positive.

## Discussion

Figure 5 shows that the image-based metrics have significant mean differences between novices’ and surgeons’ performance. Moreover, the depth constraint enhances the metrics’ performance, yielding statistically significantly different means between resident surgeons and attending surgeons. More complex movements are used to suture at depth condition, which is beneficial for distinguishing participants’ suturing skill levels. This hypothesis is further supported by Fig. 6, which shows that each group tends to have larger metric values at the depth condition than at the surface condition.

StitchLen is one of the metrics that has significantly different means for the three groups at the surface condition; however this result might be an artifact of the instructions to participants. During data collection, participants were asked to make sutures symmetric about the incision line on the membrane (see the brown circle in Fig. 1b). The geometry of the specified entry locations and incision line used in this study made the ideal StitchLen to be 13 mm, which is considered uncomfortably long from surgeons’ feedback. As shown in Fig. 6d, attending surgeons tended to make shorter stitches than the resident surgeons, suggesting that the experienced participants were more likely to disregard the instructions.

Figure 6 shows that the expert group achieves the smallest range of metric values at the depth condition for six out of eight metrics, suggesting that attending surgeons have more consistent performance as compared to resident surgeons and novices. Figure 6 also shows that the expert group has the smallest median and mean at the depth condition for metrics SweptArea, SwayLen, Range(NAngle), AVG(NAngle), and Range(TipOrth), indicating the attending surgeons have less lateral needle movement than the other participants. Furthermore, the time measurement metric (i.e., RemovalTime) suggests the attending surgeons are more efficient than the other two groups in the removal phase at the depth condition (see Fig. 6h).

Like [39], SweptArea, SwayLen, and StitchLen have significant mean differences between resident and attending surgeons at the depth condition in our dataset (see Fig. 5). Although TipPathLen shows significantly different means between the resident surgeon group and the attending surgeon group in [39], it does not show significant differences in the current dataset. In the current simulator, specular reflections from the needle occasionally cause the detected needle tip position to change between the needle midpoint and the actual needle tip. The TipPathLen metric is particularly sensitive to this noise in tip detection, which may explain the decreased effectiveness of the TipPathLen metric in the present study. The light diffuser included in the current version of SutureCoach was added to address this issue, and while it improved computer vision performance, some noise remains.

As shown in Fig. 5, Range(NAngle), AVG(NAngle), and Range(TipOrth) have significantly different means for the three groups at the depth condition. These three metrics outperform TipPathLen, in part because they are less likely to be affected by noisy needle tip detection.

Comparing Fig. 5 and 7, notice that even if two groups have statistically different means for some metrics, that does not guarantee high classification performance. For example, even though RemovalTime has *p<0.05* for the E-I comparison at the depth condition (see Fig. 5h), the corresponding ROC curve indicates RemovalTime has similar classification performance as a random classifier (see Fig. 7a). In contrast, SweptArea not only has significant mean differences for E-N and I-N comparison (see Fig. 5b) but also has the largest AUC among the eight metrics when classifying novices from surgeons at the depth condition (see Fig. 7b and c).

Figure 7 demonstrates the ROC curves for the suture classifier. ROC curves are examined for the depth condition only, since several metrics showed significantly different means between experts and intermediates only at depth, but not at surface. From Fig. 7b and c, note that SweptArea has the largest AUC between the novice and the surgeon groups. In contrast, the ROC curve of AVG(NAngle) has the smallest AUC for the novice-surgeon classification. Figure 7b and c also show that Range(TipOrth) outperforms Range(NAngle) and AVG(NAngle) for E-N and I-N classification, but AVG(NAngle) has better classification performance than Range(TipOrth) and Range(NAngle) for E-I classification (see Fig. 7a). AUC values of the metrics for the suture classifier are shown in Supplementary Table S2 in Online Resource 1.

The ROC curves for the trial classifier are shown in Fig. 8. The resolution of these ROC curves (Fig. 8) is lower than the resolution of the suture classifier ROC curves (Fig. 7), since there are twelve times as many sutures as trials. In Fig. 8c, TipPathLen has significantly larger AUC than in Fig. 7c. One reason is the noisy tip detection decreases the performance of TipPathLen in the suture classifier.

By comparing the AUC in Figs. 7 and 8, it is noticeable that the trial classifier outperforms the suture classifier, indicating the trial classifier should be used to distinguish participants’ performance. Specifically, the trial classifier with AVG(NAngle) has the best performance for E-I classification (see Fig. 8a), while the trial classifier with TipPathLen has the best performance I-N classification (see Fig. 8c). AUC values of the metrics for the trial classifier are shown in Supplementary Table S3 in Online Resource 1.

In the present work, clinical standing (i.e., attending surgeons and fellows; resident surgeons; and students) is used as a proxy for suturing skill, but suturing skill is not necessarily directly determined by clinical standing. A more objective evaluation of the suturing metrics could be obtained if an improved ground-truth assessment of suturing skill was available. For example, suturing skill could be independently assessed using expert ratings from several experienced surgeon educators.

Figure 4 shows that the threshold for splitting intermediate and expert groups is close to the peak in the year-of-experience distribution. Since years of experience is only an approximation of suturing skill, the cluster of participants close to the intermediate-expert boundary may lead to increased misclassification, even if the suturing metrics correlate well with suturing skill. This observation is supported by the results in Section 3; both the statistical results and the ROC curves show that the eight metrics are capable of distinguishing novices from surgeons, but performances decreases when comparing resident surgeons with attending surgeons. This difficulty is expected to be alleviated by introducing a better proxy for suturing skill than years of experience.

## Conclusion

This study examines eight image-based metrics for suturing skill assessment. To check the metric performance, an improved suturing simulator is used to collect a nationwide dataset with participants in different clinical standing. After analyzing the dataset using the proposed metrics, the result shows that all eight metrics have significantly different means between novices’ and surgeons’ groups. Furthermore, using the depth constraint allows image-based metrics to have significant mean differences between resident surgeons’ and attending surgeons’ groups. Also, the classification accuracy of the eight metrics is verified by ROC curves. Therefore, the eight image-based metrics are capable of suturing skill evaluation.

This work has several limitations. First, in the statistical analysis, ground-truth suturing skill was approximated by clinical standing. In the future, participants’ ground-truth suturing skill will be determined by several objective skill assessments such as expert ratings. Another limitation is that the computer vision program had inaccurate needle or thread detection on 0.5% sutures because the needle never went completely through the membrane material. To address this problem, alternative materials will be investigated for the membrane. The project focus was on suturing skill in open vascular surgery. The vast majority of resident and attending surgeon participants were recruited from vascular conferences, introducing selection bias by including residents with a preexisting interest in vascular surgery and those whose programs selected them to attend. These (and other) sampling biases may limit the representativeness of the study population and the generalizability of the findings.

## Supplementary Information

Below is the link to the electronic supplementary material.

Supplementary file 1 (docx 19 KB)

## Data Availability

The dataset presented in this article includes confidential information, so it is only available upon request. Requests to access the dataset should be directed to joseph@clemson.edu.

## References

[1] Sheahan MG, Duran C, Bismuth J. National simulation-based training of fellows: the vascular surgery example. Surg Clinics. 2015;95(4):781–90. 10.1016/j.suc.2015.04.008 26210970

[2] Mitchell EL, Sheahan MG, Schwiesow M. Simulation in vascular surgery. In: Comprehensive healthcare simulation: surgery and surgical subspecialties. 2019. pp. 327–47.

[3] Bennett KM, Kent KC, Schumacher J, Greenberg CC, Scarborough JE. Targeting the most important complications in vascular surgery. J Vasc Surg. 2017;65(3):793–803. 10.1016/j.jvs.2016.08.107 28236921

[4] Khan MS, Bann S, Darzi A, Butler P. Use of suturing as a measure of technical competence. Ann Plast Surg. 2003;50(3):304–9. 10.1097/01.sap.0000037271.26659.f4 12800910

[5] Schmiederer IS, Kearse LE, Korndorffer JR Jr, Lee E, Sgroi MD, Lee JT. Validity evidence for vascular skills assessment: The feasibility of fundamentals of vascular surgery in general surgery residency. J Surg Educ. 2021;78(6):201–9. 10.1016/j.jsurg.2021.07.009 34446383

[6] Doyle JD, Webber EM, Sidhu RS. A universal global rating scale for the evaluation of technical skills in the operating room. Am J Surg. 2007;193(5):551–5. 10.1016/j.amjsurg.2007.02.003 17434353

[7] Zia A, Sharma Y, Bettadapura V, Sarin EL, Clements MA, Essa I. Automated assessment of surgical skills using frequency analysis. In: medical image computing and computer-assisted intervention–MICCAI 2015: 18th International Conference, Munich, Germany, October 5-9, 2015, Proceedings, Part I. Springer;2015. pp. 430–438.

[8] Niitsu H, Hirabayashi N, Yoshimitsu M, Mimura T, Taomoto J, Sugiyama Y, et al. Using the objective structured assessment of technical skills (osats) global rating scale to evaluate the skills of surgical trainees in the operating room. Surg Today. 2013;43:271–5. 10.1007/s00595-012-0313-7 PMC3574562 22941345

[9] Anastakis DJ, Regehr G, Reznick RK, Cusimano M, Murnaghan J, Brown M, et al. Assessment of technical skills transfer from the bench training model to the human model. Am J Surg. 1999;177(2):167–70. 10.1016/s0002-9610(98)00327-4 10204564

[10] Sidhu RS, Park J, Brydges R, MacRae HM, Dubrowski A. Laboratory-based vascular anastomosis training: a randomized controlled trial evaluating the effects of bench model fidelity and level of training on skill acquisition. J Vasc Surg. 2007;45(2):343–9. 10.1016/j.jvs.2006.09.040 17264015

[11] Datta V, Mackay S, Mandalia M, Darzi A. The use of electromagnetic motion tracking analysis to objectively measure open surgical skill in the laboratory-based model. J Am Coll Surg. 2001;193(5):479–85. 10.1016/s1072-7515(01)01041-9 11708503

[12] Bann SD, Khan MS, Darzi AW. Measurement of surgical dexterity using motion analysis of simple bench tasks. World J Surg. 2003;27:390–4. 10.1007/s00268-002-6769-7 12658479

[13] Datta V, Chang A, Mackay S, Darzi A. The relationship between motion analysis and surgical technical assessments. Am J Surg. 2002;184(1):70–3. 10.1016/s0002-9610(02)00891-7 12135725

[14] Dubrowski A, Sidhu R, Park J, Carnahan H. Quantification of motion characteristics and forces applied to tissues during suturing. Am J Surg. 2005;190(1):131–6. 10.1016/j.amjsurg.2005.04.006 15972186

[15] Singapogu RB, Kavathekar T, Eidt JF, Groff RE, Burg TC. A novel platform for assessment of surgical suturing skill: Preliminary results, MMVR; 2016. pp. 375–8. 27046608

[16] Yamaguchi S, Yoshida D, Kenmotsu H, Yasunaga T, Konishi K, Ieiri S, et al. Objective assessment of laparoscopic suturing skills using a motion-tracking system. Surg Endosc. 2011;25:771–5. 10.1007/s00464-010-1251-3 21072671

[17] Uemura M, Tomikawa M, Miao T, Souzaki R, Ieiri S, Akahoshi T, et al. Feasibility of an ai-based measure of the hand motions of expert and novice surgeons. Computational and mathematical methods in medicine. 2018. 10.1155/2018/9873273 PMC5857335 29686724

[18] Sharon Y, Jarc AM, Lendvay TS, Nisky I. Rate of orientation change as a new metric for robot-assisted and open surgical skill evaluation. IEEE Trans Med Robot Bionics. 2021;3(2):414–25.

[19] Shayan AM, Singh S, Gao J, Groff RE, Bible J, Eidt JF, et al. Measuring hand movement for suturing skill assessment: a simulation-based study. Surgery. 2023;174(5):1184–92. 10.1016/j.surg.2023.07.007 PMC10592328 37597999

[20] Shayan AM, Hitchcock DB, Singh S, Gao J, Groff RE, Singapogu RB. Functional data analysis of hand rotation for open surgical suturing skill assessment. IEEE J Biomed Health Inform. 2024 10.1109/JBHI.2024.3496122 PMC12083055 39527415

[21] Singh SP, Shayan AM, Gao J, Bible J, Groff RE, Singapogu R. Objective and automated quantification of instrument handling for open surgical suturing skill assessment: A simulation-based study. IEEE Open J Eng Med Biol. 2024 10.1109/OJEMB.2024.3402393 PMC11268937 39050974

[22] Trejos AL, Patel RV, Malthaner RA, Schlachta CM. Development of force-based metrics for skills assessment in minimally invasive surgery. Surg Endosc. 2014;28:2106–19. 10.1007/s00464-014-3442-9 24519030

[23] Richards C, Rosen J, Hannaford B, Pellegrini C, Sinanan M. Skills evaluation in minimally invasive surgery using force/torque signatures. Surg Endosc. 2000;14:791–8. 10.1007/s004640000230 11000356

[24] Horeman T, Rodrigues SP, Jansen F-W, Dankelman J, Dobbelsteen JJ. Force measurement platform for training and assessment of laparoscopic skills. Surg Endosc. 2010;24:3102–8. 10.1007/s00464-010-1096-9 PMC2982957 20464416

[25] Singh SP, Shayan AM, Gao J, Biblev J, Groff RE, Singapogu R. Evaluating smoothness of force for surgical skill assessment. In: 2024 46th Annual international conference of the IEEE engineering in medicine and biology society (EMBC). IEEE; 2024. pp. 1–4. 10.1109/EMBC53108.2024.10781655 40039007

[26] Frischknecht AC, Kasten SJ, Hamstra SJ, Perkins NC, Gillespie RB, Armstrong TJ, et al. The objective assessment of experts’ and novices’ suturing skills using an image analysis program. Acad Med. 2013;88(2):260–4. 10.1097/ACM.0b013e31827c3411 23269303

[27] Islam G, Kahol K, Li B, Smith M, Patel VL. Affordable, web-based surgical skill training and evaluation tool. J Biomed Inform. 2016;59:102–14. 10.1016/j.jbi.2015.11.002 26556643

[28] Islam G, Kahol K, Li B. Development of a computer vision application for surgical skill training and assessment. Bio-informatic systems processing and applications. 2013; 191.

[29] Pérez-Escamirosa F, Alarcón-Paredes A, Alonso-Silverio GA, Oropesa I, Camacho-Nieto O, Lorias-Espinoza D, et al. Objective classification of psychomotor laparoscopic skills of surgeons based on three different approaches. Int J Comput Assist Radiol Surg. 2020;15(1):27–40. 10.1007/s11548-019-02073-2 31605351

[30] Gao J, Shayan AM, Singh SP, Bible J, Singapogu R, Groff RE. Surgical suturing skill assessment using estimated hand roll angle from a deep-learning computer vision algorithm. In: 2024 46th annual international conference of the IEEE engineering in medicine and biology society (EMBC). IEEE; 2024. pp. 1–6. 10.1109/EMBC53108.2024.10782514 40039188

[31] Funke I, Mees ST, Weitz J, Speidel S. Video-based surgical skill assessment using 3d convolutional neural networks. Int J Comput Assist Radiol Surg. 2019;14:1217–25. 10.1007/s11548-019-01995-1 31104257

[32] Khalid S, Goldenberg M, Grantcharov T, Taati B, Rudzicz F. Evaluation of deep learning models for identifying surgical actions and measuring performance. JAMA Netw Open. 2020;3(3):201664–201664. 10.1001/jamanetworkopen.2020.1664 PMC12124734 32227178

[33] Liu D, Li Q, Jiang T, Wang Y, Miao R, Shan F, Li Z. Towards unified surgical skill assessment. In: Proceedings of the IEEE/CVF conference on computer vision and pattern recognition. 2021. pp. 9522–9531.

[34] Soleymani A, Asl AAS, Yeganejou M, Dick S, Tavakoli M, Li X. Surgical skill evaluation from robot-assisted surgery recordings. In: 2021 International symposium on medical robotics (ISMR). IEEE; 2021. pp. 1–6.

[35] Wang Y, Dai J, Morgan TN, Elsaied M, Garbens A, Qu X, et al. Evaluating robotic-assisted surgery training videos with multi-task convolutional neural networks. J Robot Surg. 2021;1–9. 10.1007/s11701-021-01316-2 34709538

[36] Kil I, Eidt JF, Groff RE, Singapogu RB. Assessment of open surgery suturing skill: Simulator platform, force-based, and motion-based metrics. Front Med. 2022;9:897219. 10.3389/fmed.2022.897219 PMC9468321 36111107

[37] Kil I, Eidt JF, Singapogu RB, Groff RE. Assessment of open surgery suturing skill: Image-based metrics using computer vision. J Surg Educ. 2024;(accepted). 10.1016/j.jsurg.2024.03.020 PMC11181522 38749810

[38] Kil I, Groff RE, Singapogu RB. Surgical suturing with depth constraints: Image-based metrics to assess skill. In: 40th annual international conference of the IEEE engineering in medicine and biology society (EMBC). IEEE; 2018. pp. 4146–9. 10.1109/EMBC.2018.8513266 30441268

[39] Kil I. Development and preliminary validation of image-enabled process metrics for assessment of open surgery suturing skill. Clemson University; 2019.(PhD thesis).

[40] Gao J. Computer vision algorithms for assessment of surgical suturing skill using hand and needle motion. Clemson University; 2024.(PhD thesis).

[41] Dosis A, Aggarwal R, Bello F, Moorthy K, Munz Y, Gillies D, et al. Synchronized video and motion analysis for the assessment of procedures in the operating theater. Arch Surg. 2005;140(3):293–9. 10.1001/archsurg.140.3.293 15781796

[42] Sherris DA, Kern EB. Essent Surg Skills. Philadelphia, PA: Saunders; 2004.

[43] Semer NB, Adler-Lavan M. Practical Plastic Surgery for Nonsurgeons. Philadelphia, PA: Hanley & Belfus Philadelphia; 2001.

[44] Jackson RC, Çavuşoğlu MC. Needle path planning for autonomous robotic surgical suturing. In: 2013 IEEE International conference on robotics and automation. IEEE; 2013. pp. 1669–75. 10.1109/ICRA.2013.6630794 PMC3966119 24683500

[45] Kavathekar T, Kil I, Groff RE, Burg TC, Eidt JF, Singapogu RB. Towards quantifying surgical suturing skill with force, motion and image sensor data. In: 2017 IEEE EMBS International conference on biomedical & health informatics (BHI). 2017. pp. 169–172. IEEE.

[46] Jackson RC, Çavuşoğlu MC. Modeling of needle-tissue interaction forces during surgical suturing. In: 2012 IEEE international conference on robotics and automation. IEEE; 2012. p. 4675–80. 10.1109/ICRA.2012.6224756 PMC3966136 24683499

[47] Treil L, Neumann N, Chanes N, Lejay A, Bourcier T, Bismuth J, et al. Objective evaluation of clock face suture using the objective structured assessment of technical skill (osats) checklist. EJVES Vascular Forum. 2022;57:5–11. 10.1016/j.ejvsvf.2022.10.001 PMC9664359 36388464

[48] Ferri C, Hernández-Orallo J, Flach PA. A coherent interpretation of auc as a measure of aggregated classification performance. In: proceedings of the 28th international conference on machine learning (ICML-11). 2011. pp. 657–64.

[49] Hanley JA, McNeil BJ. The meaning and use of the area under a receiver operating characteristic (roc) curve. Radiology. 1982;143(1):29–36. 10.1148/radiology.143.1.7063747 7063747

