# Supplementary material for: Developing and evaluating image-based metrics for open surgical suturing skill assessment
Source: Global Surg Educ. 2026 Oct 13;5(1):196. doi: 10.1007/s44186-026-00596-2 (PMC13661727; doi:10.1007/s44186-026-00596-2)
Supplement: Supplementary file 1 [file 44186_2026_596_MOESM1_ESM.docx]

**SUPPLEMENTARY MATERIAL**

Table S1. Pairwise comparisons for skill assessment metrics among three groups: Expert (E), Intermediate (I), and Novice (N). Each table cell shows data in the format: mean difference (standard error, p-value). Table cells with p<0.05 is highlighted in bold. The table shows results at surface and depth conditions

|  | E vs. I (surface) | E vs. N (surface) | I vs. N (surface) | E vs. I (depth) | E vs. N (depth) | I vs. N (depth) |
| --- | --- | --- | --- | --- | --- | --- |
| TipPathLen | 0.72(0.86, 0.4) | **-14.4(2.07, <0.01)** | **-15.2(2.08, <0.01)** | 0.93(1.75, 0.6) | **-32(3.49, <0.01)** | **-32.9(3.43, <0.01)** |
| SweptArea | -1.23(1.06, 0.24) | **-21.2(1.58, <0.01)** | **-20(1.64, <0.01)** | **-6.44(1.47, <0.01)** | **-36.1(2.07, <0.01)** | **-29.7(2.11, <0.01)** |
| SwayLen | -0.04(0.04, 0.26) | **-0.58(0.05, <0.01)** | **-0.54(0.05, <0.01)** | **-0.26(0.05, <0.01)** | **-0.86(0.06, <0.01)** | **-0.6(0.06, <0.01)** |
| StitchLen | **-0.38(0.12, <0.01)** | **-1.44(0.13, <0.01)** | **-1.06(0.13, <0.01)** | **-0.59(0.12, <0.01)** | **-1.64(0.13, <0.01)** | **-1.05(0.13, <0.01)** |
| Range(NAngle) | -0.44(1.28, 0.73) | **-16(1.56, <0.01)** | **-15.6(1.56, <0.01)** | **-5.49(1.71, <0.01)** | **-27(2.01, <0.01)** | **-21.5(1.99, <0.01)** |
| AVG(NAngle) | -0.23(0.31, 0.45) | **-3.86(0.36, <0.01)** | **-3.63(0.37, <0.01)** | **-3.11(0.46, <0.01)** | **-6.13(0.5, <0.01)** | **-3.02(0.53, <0.01)** |
| Range(TipOrth) | 0.05(0.09, 0.54) | **-1.41(0.12, <0.01)** | **-1.46(0.12, <0.01)** | **-0.6(0.14, <0.01)** | **-2.77(0.17, <0.01)** | **-2.17(0.17, <0.01)** |
| RemovalTime | **-0.29(0.11, <0.01)** | **-1.63(0.14, <0.01)** | **-1.33(0.14, <0.01)** | **-0.38(0.11, <0.01)** | **-1.86(0.16, <0.01)** | **-1.48(0.17, <0.01)** |

Table S2. Suture classifier: Area under curve (AUC) for skill assessment metrics among three groups: Expert (E), Intermediate (I), and Novice (N). The largest value in each column is highlighted in bold. The table only shows results at depth condition

|  | E vs. I  (depth) | E vs. N  (depth) | I vs. N  (depth) |
| --- | --- | --- | --- |
| TipPathLen | 0.501 | 0.69 | 0.698 |
| SweptArea | 0.581 | **0.76** | **0.704** |
| SwayLen | 0.561 | 0.707 | 0.655 |
| StitchLen | 0.565 | 0.684 | 0.639 |
| Range(NAngle) | 0.566 | 0.696 | 0.649 |
| AVG(NAngle) | **0.599** | 0.668 | 0.58 |
| Range(TipOrth) | 0.544 | 0.718 | 0.688 |
| RemovalTime | 0.527 | 0.67 | 0.643 |

Table S3. Trial classifier: Area under curve (AUC) for skill assessment metrics among three groups: Expert (E), Intermediate (I), and Novice (N). The largest value in each column is highlighted in bold. The table only shows results at depth condition

|  | E vs. I  (depth) | E vs. N  (depth) | I vs. N  (depth) |
| --- | --- | --- | --- |
| TipPathLen | 0.482 | 0.806 | **0.838** |
| SweptArea | 0.609 | **0.863** | 0.783 |
| SwayLen | 0.632 | 0.811 | 0.725 |
| StitchLen | 0.595 | 0.755 | 0.703 |
| Range(NAngle) | 0.615 | 0.782 | 0.72 |
| AVG(NAngle) | **0.657** | 0.731 | 0.615 |
| Range(TipOrth) | 0.604 | 0.836 | 0.777 |
| RemovalTime | 0.547 | 0.709 | 0.652 |
